# Retraction of: Differentiation Between Humic and Non-Humic Substances Using Alkaline Extraction and Ultraviolet Spectroscopy

**DOI:** 10.1093/jaoacint/qsae102

**Published:** 2025-01-20

This is a retraction of: Lawrence Mayhew, Amit Pratap Singh, Peng Li, E Michael Perdue, Differentiation Between Humic and Non-Humic Substances Using Alkaline Extraction and Ultraviolet Spectroscopy, *Journal of AOAC INTERNATIONAL*, Volume 106, Issue 3, May-June 2023, Pages 748–759, https://doi.org/10.1093/jaoacint/qsad001

The corresponding author contacted the Journal and Association in November 2024, requesting the article be retracted because the article contains a flawed procedure for calibration of the spectrometer.

Within the Experimental Procedure section of the article, Step 13 of the *Scan* sub-section:

1. Did not sufficiently detail the Quality Control Material (QCM), including the need for a minimum of 50% humic acids and 2% hydrophobic fulvic acids concentration, with a total dissolved organic matter content of 30 mg kg-1.
2. Omitted calibration data, specifically that the UV absorbance data from 290 to 330 nm must be within ±2 standard deviations of the population data of the QCM, as well as what action to take if the results exceeded the ±2 standard deviations.
3. Did not specify the importance of the Humic Dashboard spreadsheet nor provide guidance on next steps.

The effect of the omissions above resulted in a lack of confidence in the data itself and in the conclusions drawn from it.

The editors and corresponding author have agreed to the retraction.

